# Node deposit size index as morphometric marker of intranodal tumour burden in node-positive oral cancer: a hypothesis-generating cohort study

**DOI:** 10.3389/fonc.2026.1923931

**Published:** 2026-09-07

**Authors:** Karthik N. Rao, Teertha Shetty, Shanthi Velusamy, Prajwal Dange, Sreeram MP, B. S. Srinath

**Affiliations:** 1 Department of Head and Neck Surgery and Oncology, Sri Shankara Cancer Hospital, Bangalore, Karnataka, India; 2 Department of Pathology, Sri Shankara Cancer Hospital, Bangalore, Karnataka, India; 3 Department of Surgical Oncology, Sri Shankara Cancer Hospital, Bangalore, Karnataka, India

**Keywords:** lymphatic metastasis, morphometry, neoplasm staging, oral cancer, prognosis

## Abstract

**Background:**

Cervical nodal metastasis critically influences prognosis in oral squamous cell carcinoma (OSCC), yet current staging does not quantify the degree of metastatic replacement within lymph nodes. This study evaluated morphometric index Node Deposit Size Index (NDSI) to assess their association with pathological aggressiveness and survival.

**Methods:**

An ambispective cohort of 104 surgically treated, node-positive OSCC patients was analysed. Maximum metastatic deposit diameter and lymph node diameter were measured on routine H&E slides. NDSI correlated with clinicopathological variables, extranodal extension (ENE), lymph node density (LND), and survival using non-parametric tests, Spearman correlation, Kaplan–Meier analysis, and ROC curves.

**Results:**

ENE-positive nodes demonstrated higher NDSI than ENE-negative nodes (86.9% *vs* 43.8%; *p* < 0.001). NDSI increased across pathological nodal stage (pN), from 31.9% in pN1 to 87.8% in pN3b (*p* < 0.001), and across overall stage, reaching 87.9% in Stage IVB (*p* < 0.001). Higher NDSI was associated with lymphovascular invasion (LVI) (72.0% *vs* 45.4%; *p* < 0.001) and perineural invasion (69.5% *vs* 50.7%; *p* = 0.024). NDSI correlated with LND (Spearman ρ=0.43, *p* < 0.001). ROC analysis identified an NDSI cutoff of 73.3% for predicting ENE (AUC = 0.854; not internally validated) and a cutoff of 60% for predicting 1-year mortality (AUC = 0.708; not internally validated). On multivariable linear regression (R² = 0.524), LVI and pN (pN2a, pN2b, and pN3b) were independently associated with higher NDSI, whereas the remaining clinicopathological variables were not independently significant.

**Conclusion:**

NDSI correlate with adverse pathology and early mortality. However, the association between NDSI and ENE is not fully independent of pathological N-stage, and reproducibility, independent prognostic value, and cutoff stability require external validation before clinical use.

## Introduction

Oral squamous cell carcinoma (OSCC) frequently presents with cervical lymph node metastasis, which remains one of the most important determinants of survival, recurrence, and adjuvant treatment selection (1). Pathological nodal assessment forms a major component of contemporary risk stratification systems in OSCC (2).

Current American Joint Committee on Cancer (AJCC) nodal staging incorporates nodal number, size, laterality, and extranodal extension (ENE) (3). Although these variables are clinically validated, they do not directly quantify the extent of metastatic replacement within the involved lymph node. Metastatic nodes demonstrate substantial histological heterogeneity. Some nodes contain small metastatic foci with preserved nodal architecture, whereas others demonstrate near complete replacement by tumour despite similar nodal dimensions (4).

Several nodal metrics including lymph node ratio (LNR) and lymph node density (LND) have therefore been evaluated to improve prognostic stratification. However, these parameters depend on neck dissection yield and total nodal counts rather than direct assessment of intranodal tumour burden (5).

Quantification of metastatic occupancy within the lymph node has received limited evaluation in OSCC. Two nodes of the same diameter can differ in the proportion of metastatic deposit versus preserved nodal parenchyma. The degree of nodal replacement by tumour may serve as an objective morphometric surrogate of biological aggressiveness and progressive nodal destruction, although this hypothesis has not been directly tested in OSCC and is explored here for the first time (6, 7).

To address this gap, we devised morphometric index derived from measurements already routinely recorded on histopathology. The Node Deposit Size Index (NDSI) provides a linear morphometric estimate of the relative extent of metastatic replacement within an involved lymph node using routinely measured pathological dimensions. The ratio of maximum metastatic deposit diameter (MDD) to largest lymph node diameter (LyND). NDSI is intended as supplementary, quantitative descriptors of intranodal tumour burden, and not as substitutes for existing staging frameworks.

The aim of the present study was to explore the association of NDSI with clinicopathological parameters, pathological staging, extranodal extension, lymph node density, tumour infiltrating lymphocytes, and survival outcomes in node-positive OSCC.

## Subjects and methods

### Study design and setting

An ambispective observational cohort study was conducted at a tertiary head and neck oncology centre between January 2024 and May 2025. The study included retrospective and prospective accrual of patients with histopathologically confirmed OSCC who underwent primary surgical treatment with neck dissection.

Institutional ethics committee approval was obtained vide Approval No. SSCHRC/IEC27/229. Clinical records, operative details, histopathological reports, and archived haematoxylin and eosin-stained slides were reviewed.

### Study population

This study included patients with histopathologically confirmed oral squamous cell carcinoma (OSCC) who underwent primary surgical treatment with neck dissection at our centre between January 2024 and May 2025. Eligible patients demonstrated pathologically confirmed metastatic cervical lymph node disease and had a minimum follow-up duration of 1 year, or a documented earlier death event. Patients were excluded if they presented with node-negative disease, non-squamous histology, inadequate histopathological material, or incomplete morphometric nodal measurements.

### Histopathological evaluation

Histopathological slides were reviewed for assessment of primary tumour and nodal characteristics. The following clinicopathological and morphometric parameters were documented: age, sex, primary tumour site and subsite, pathological T stage, pathological N stage, overall pathological stage, tumour differentiation, worst pattern of invasion (WPOI), pattern of perineural invasion (PPOI), perineural invasion (PNI), lymphovascular invasion (LVI), depth of invasion (DOI), tumour infiltrating lymphocytes (TILs), neoadjuvant chemotherapy status, ENE, ENE size, largest involved neck node level, total lymph node yield, number of metastatic lymph nodes, LND, LyND, MDD, NDSI, survival duration, and survival status. Staging was performed according to AJCC 8th edition criteria.

**Figure 1:**
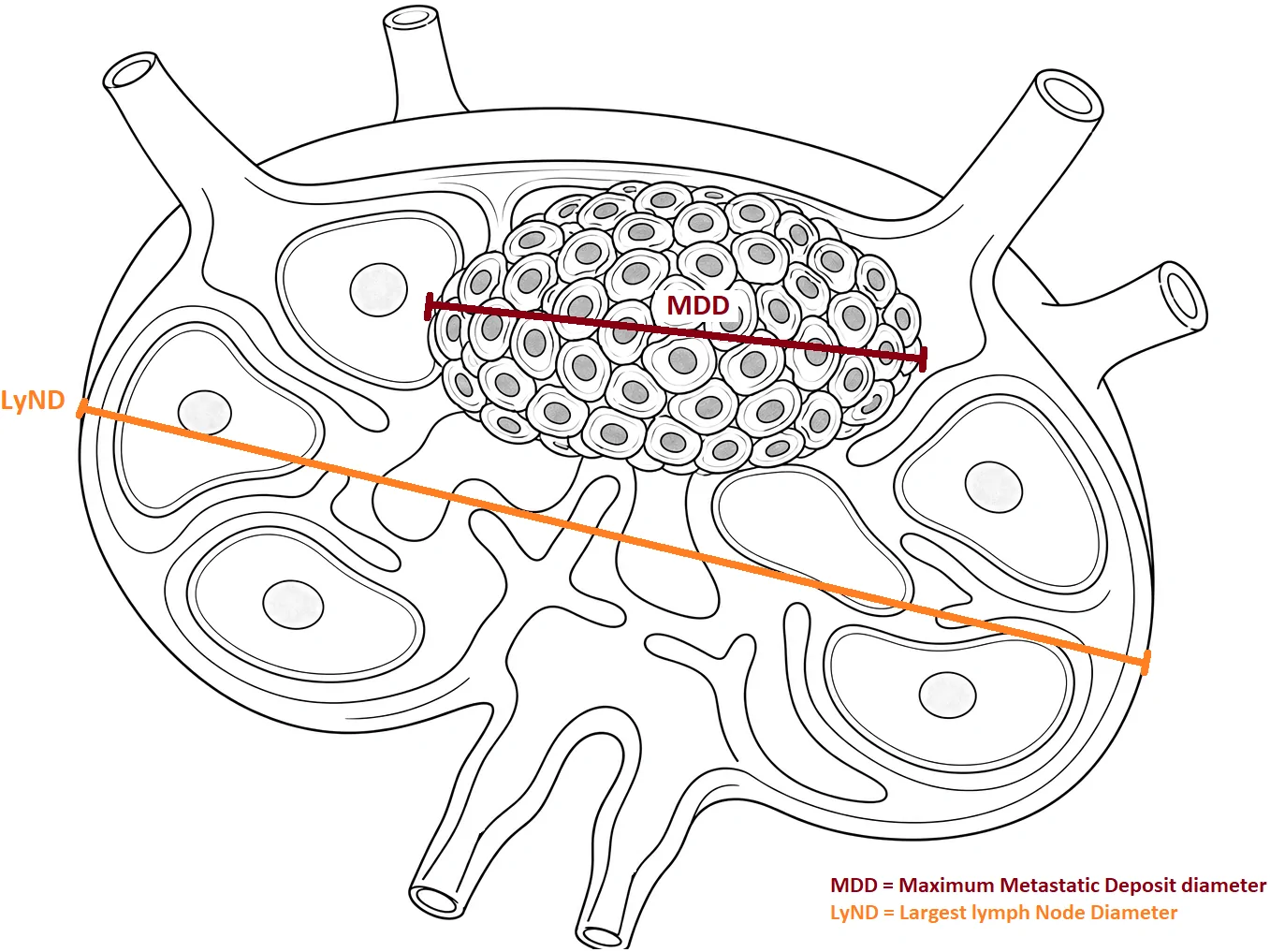
Metastatic deposit within a metastatic lymph node.

**Figure 2:**
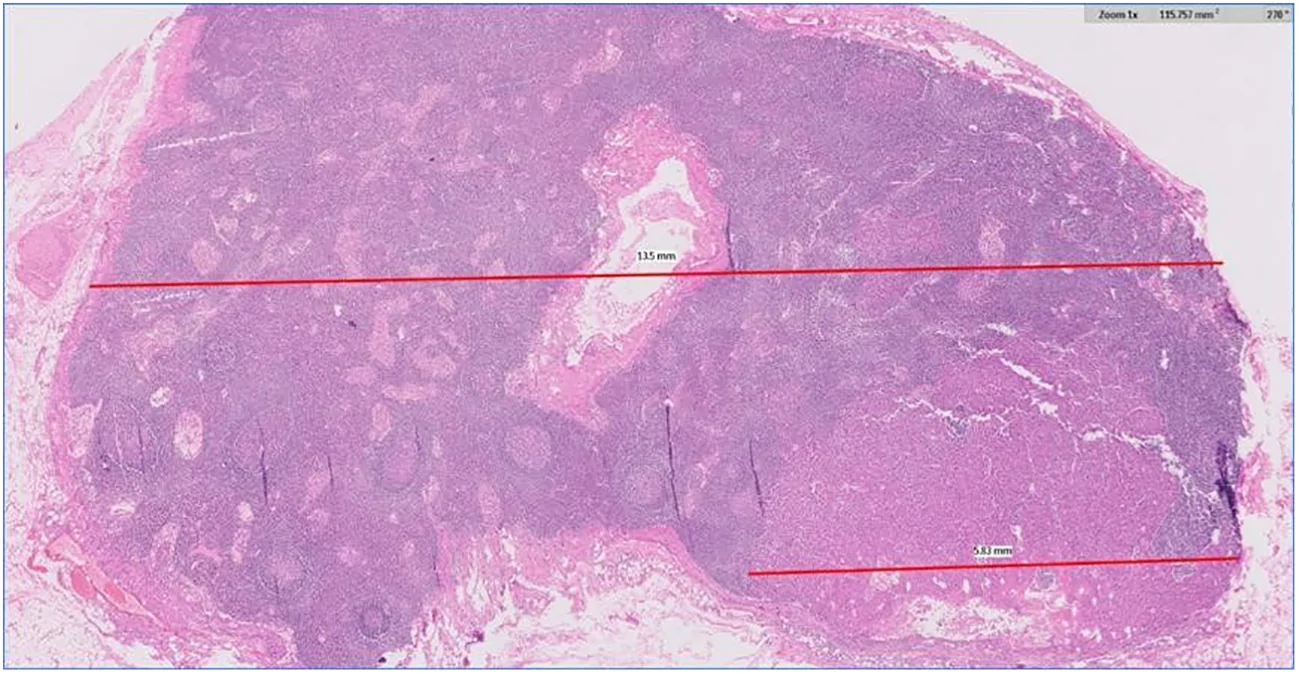
Haematoxylin and eosin-stained slide depicting the metastatic deposit within the metastatic lymph node.

Morphometric analysis was performed using metastatic lymph node sections on routine haematoxylin and eosin-stained slides (**Figures 1**, **2**). LyND was defined as the maximum diameter of the involved lymph node including both metastatic tumour and residual nodal tissue. MDD was defined as the maximum diameter of metastatic tumour deposit within the lymph node, excluding uninvolved nodal tissue. Measurements were obtained directly from histopathological sections. Linear diameter measurements were intentionally selected because they can be performed rapidly on routine haematoxylin and eosin-stained sections without dedicated image-analysis software or volumetric reconstruction. Although true intranodal occupancy is a three-dimensional volumetric property, the ratio of maximal linear diameters provides a reasonable first-order approximation under the assumption of roughly proportional growth in metastatic deposits relative to the host node, an assumption consistent with approaches used for analogous linear ratios in other solid tumour staging systems (e.g., tumour diameter as a surrogate for tumour volume in T-staging). This approach was chosen to maximise clinical applicability and facilitate integration into routine pathology workflows. NDSI was calculated as the ratio of the maximum metastatic deposit diameter to the maximum diameter of the involved lymph node, expressed as a percentage. This ratio was selected because it normalizes metastatic deposit size to lymph node size, thereby providing a simple linear morphometric estimate of the relative extent of metastatic replacement within an involved lymph node. NDSI is not intended to represent the true area or volumetric fraction occupied by tumour, but rather a practical first-order approximation derived from routine two-dimensional histopathological measurements. LND was calculated as the ratio of metastatic lymph nodes to total nodal yield.

When multiple metastatic deposits were present within a single lymph node, the largest contiguous metastatic deposit was measured for calculation of NDSI. For nodes with extensive or matted replacement where individual deposit boundaries could not be distinguished, the entire region of confluent tumour was treated as a single deposit. Smaller satellite deposits within the same node were not incorporated into the index, which may underestimate true intranodal tumour burden in multifocal disease. Measurements were performed on a single haematoxylin and eosin-stained section per node, selected as the section showing maximal cross-sectional tumour diameter, in keeping with conventional practice for reporting maximum tumour dimension in routine histopathology.

Measurements were performed by a single experienced head and neck pathologist using the predefined node-selection protocol described above. Measurements were not performed in a blinded fashion with respect to clinicopathological variables or outcomes, and this has been acknowledged as a potential source of measurement bias. Because the same measurements (MDD, LyND) were used to derive NDSI and also contributed to ENE assessment and N-staging, non-blinded single-observer measurement introduces a risk of incorporation bias, particularly for ENE-correlated analyses. Formal interobserver agreement was not assessed; reproducibility across independent, blinded observers remains an important objective for future validation studies.

NDSI was calculated on a per-patient basis. In patients with multiple metastatic lymph nodes, measurements were obtained from the largest lymph node containing the largest metastatic deposit (maximum MDD), and the corresponding lymph node diameter (LyND) from that same node was used for NDSI calculation. This predefined selection rule was applied uniformly across all patients.

### Survival analysis

Overall survival was calculated from date of treatment to date of death or last follow-up. Patients alive at last follow-up were censored. Kaplan–Meier survival curves were generated for NDSI and ENE-positive versus ENE-negative disease. Survival distributions were compared using the log-rank test. Receiver operating characteristic (ROC) analysis was additionally performed to determine the optimal NDSI cutoff associated with death within 1 year.

### Statistical analysis

Statistical analyses were performed using Python-based statistical libraries. Normality assessment was performed using the Shapiro–Wilk test and demonstrated non-normal distribution of NDSI. Binary variables were analysed using the Mann–Whitney U test, while multi-category variables were analysed using the Kruskal–Wallis H test. Correlations were analysed using Spearman rank correlation. Survival analysis was performed using Kaplan–Meier methodology with log-rank testing. ROC analyses were performed using Youden index-derived optimal cutoff values with area under the curve (AUC) estimation.

A multivariable linear regression model was performed with NDSI (%) as the dependent variable. Independent variables included histological differentiation, WPOI, PPOI, PNI, LVI, TILs, NACT, pathological T category, and pathological N category. Variables were entered simultaneously into the multivariable linear regression model, with categorical variables included using appropriate reference categories. Regression coefficients (β), 95% confidence intervals (CI), and P values were reported.

Continuous variables were summarized using means and medians with interquartile ranges. Subgroups containing fewer than five observations were interpreted descriptively and omitted from inferential testing due to limited statistical power. No formal sample size or power calculation was performed prior to data collection, as this was a hypothesis-generating exploratory study; the final sample size was determined by the number of eligible patients accrued during the study period. Because of the exploratory nature of this study, multiple subgroup analyses were conducted without formal adjustments for multiplicity; individual p-values should be interpreted as hypothesis-generating. A p-value <0.05 was considered statistically significant.

## Results

### Cohort characteristics

Among 229 patients who underwent primary surgery for oral squamous cell carcinoma (OSCC) between January 2024 and May 2025, 109 had pathologically confirmed cervical lymph node metastases. Five patients were excluded because of incomplete morphometric measurements, leaving 104 node-positive patients for analysis.

Histopathological data were complete for differentiation, pathological T category, pathological N category, overall stage, depth of invasion (DOI), tumour-infiltrating lymphocytes (TILs), perineural invasion (PNI), lymphovascular invasion (LVI), extranodal extension (ENE), neoadjuvant chemotherapy (NACT), and survival outcomes. ENE thickness measurements were available for all 49 ENE-positive patients.

### Histopathological predictors of nodal tumour burden

Mean NDSI increased from 48.0% in well-differentiated SCC to 78.1% in poorly differentiated SCC and 83.3% in spindle cell SCC; this trend did not reach statistical significance (Kruskal–Wallis H test, p=0.232) (**Table 1**).

**Table 1:** NDSI *vs* histopathological factors.

Variable	Category	N	NDSI (Mean ± SD)	Median (Q1–Q3)	Statistical test	*p* value
Differentiation/Histologic Subtype	Well differentiated	3	48.0 ± 27.7	32.0 (32.0–56.0)	Kruskal–Wallis H	0.232
	Moderately differentiated	88	62.4 ± 34.7	64.0 (24.8–100.0)		
	Poorly differentiated	10	78.1 ± 28.8	100.0 (59.5–100.0)		
	Spindle Cell SCC	3	83.3 ± 28.9	100.0 (75.0–100.0)		
WPOI*	WPOI-3	9	70.7 ± 37.5	80.0 (37.5–100.0)	Kruskal–Wallis H	0.163
	WPOI-4	52	57.2 ± 35.1	52.3 (23.3–100.0)		
	WPOI-5	43	71.0 ± 31.1	81.3 (48.7–100.0)		
PPOI*	Pattern 3	11	68.4 ± 38.8	80.0 (32.0–100.0)	Kruskal–Wallis H	0.440
	Pattern 4	59	60.2 ± 34.5	60.0 (25.0–100.0)		
	Pattern 5	34	69.5 ± 31.8	80.6 (40.0–100.0)		
PNI	No	30	50.7 ± 38.6	36.0 (20.0–84.0)	Mann–Whitney U	0.024
	Yes	74	69.5 ± 30.7	80.0 (44.0–100.0)		
LVI	No	31	45.4 ± 34.5	35.0 (16.8–80.0)	Mann–Whitney U	<0.001
	Yes	73	72.0 ± 30.8	81.3 (50.0–100.0)		
TILs	Low (<20%)	8	76.3 ± 36.5	84.0 (53.5–100.0)	Kruskal–Wallis H	0.071
	Intermediate (20–50%)	73	66.7 ± 33.0	80.0 (35.0–100.0)		
	High (>50%)	23	51.7 ± 33.0	48.0 (18.0–82.9)		
NACT	No	87	61.6 ± 33.9	60.0 (24.0–71.5)	Mann–Whitney U	0.118
	Yes	17	76.8 ± 33.1	100.0 (40.0–100.0)		

*Cases with unavailable/missing data were excluded from subgroup-specific inferential testing. Comparisons involving very small subgroups, including well-differentiated SCC (n=3), spindle cell SCC (n=3), pT4b (n=2), pN2c (n=1), and ENE ≤2 mm (n=4), should be interpreted cautiously because of limited sample size.

Higher WPOI and PPOI grades were associated with numerically increased NDSI, but neither WPOI (p=0.163) nor PPOI (p=0.440) reached statistical significance (**Table 1**).

PNI was associated with significantly greater nodal tumour burden. PNI-positive tumours demonstrated significantly higher mean NDSI than PNI-negative tumours (69.5% *vs* 50.7%; p=0.024) (**Table 1**).

Similarly, lvi demonstrated association with nodal tumour burden. LVI-positive tumours exhibited significantly higher mean NDSI than LVI-negative tumours (72.0% *vs* 45.4%; p<0.001) (**Table 1**).

Although tumours with low TIL density (<20%) demonstrated progressively higher NDSI values than those with high TIL density (>50%) (76.3% *vs* 51.7%), this trend did not reach statistical significance (p=0.071) (**Table 1**).

Patients who received neoadjuvant chemotherapy demonstrated numerically higher NDSI than those treated with upfront surgery (76.8% *vs* 61.6%); however, this difference was not statistically significant (p=0.118) (**Table 1**).

### Association with tumour stage and nodal characteristics

Mean NDSI ranged from 55.9% in pT2 disease to 100% in the two pT4b patients (n=2, descriptive only); differences across pathological T categories were not statistically significant (p=0.377) (**Table 2**).

**Table 2:** NDSI *vs* tumour stage and nodal characteristics.

Variable	Category	N	NDSI (mean ± SD)	NDSI median (Q1–Q3)	Statistical test	P-value
T Category	T0	1	32.0	32.0 (32.0–32.0)	Kruskal–Wallis H test	0.377
	T1	8	58.0 ± 43.6	70.0 (11.0–100.0)		
	T2	24	55.9 ± 31.7	49.0 (24.8–88.8)		
	T3	26	67.6 ± 33.0	68.7 (50.0–100.0)		
	T4a	43	66.7 ± 34.5	80.0 (34.4–100.0)		
	T4b†	2	100.0 ± 0.0	100.0 (100.0–100.0)		
Pathological N Category	pN1	29	31.9 ± 24.3	20.0 (12.0–45.0)	Kruskal–Wallis H test	<0.001
	pN2a	5	72.6 ± 29.6	80.0 (42.9–100.0)		
	pN2b	23	57.9 ± 31.2	60.0 (28.5–83.4)		
	pN2c†	1	8.0	8.0 (8.0–8.0)		
	pN3b	46	87.8 ± 20.0	100.0 (80.3–100.0)		
Overall Stage	Stage III	12	28.1 ± 18.4	27.7 (11.0–43.3)	Kruskal–Wallis H test	<0.001
	Stage IVA	47	50.5 ± 32.9	44.4 (20.0–80.0)		
	Stage IVB	45	87.9 ± 19.2	100.0 (80.0–100.0)		
Highest Involved Neck Level	Level I	46	62.7 ± 32.9	64.0 (35.4–100.0)	Kruskal–Wallis H test	0.006
	Level II	9	86.3 ± 19.5	100.0 (70.0–100.0)		
	Level III	6	91.7 ± 20.4	100.0 (100.0–100.0)		
	Level IV	32	50.6 ± 32.5	50.0 (23.3–80.8)		
	Level V	11	74.5 ± 40.7	100.0 (51.3–100.0)		
Depth of Invasion (DOI)	<5 mm	8	54.6 ± 36.6	52.5 (27.0–85.0)	Kruskal–Wallis H test	0.671
	5–10 mm	34	63.4 ± 33.7	60.0 (36.3–100.0)		
	>10 mm	62	65.7 ± 34.3	80.0 (32.8–100.0)		

†Categories containing fewer than five observations should be interpreted cautiously.

Mean NDSI progressively increased from 31.9% in pN1 to 87.8% in pN3b disease (p<0.001) (**Table 2**). A comparable stepwise relationship was observed with overall pathological stage, with mean NDSI increasing from 28.1% in Stage III to 87.9% in Stage IVB disease (p<0.001) (**Table 2**).

The highest involved neck level was significantly associated with nodal tumour burden (p=0.006). Mean NDSI ranged from 50.6% for Level IV disease to 91.7% for Level III disease; however, interpretation of Levels II, III and V should be made cautiously because of small subgroup sizes (**Table 2**).

Increasing DOI demonstrated a numerical increase in NDSI (>10 mm: 65.7%) compared with DOI <5 mm (54.6%), although this difference was not statistically significant (p=0.671) (**Table 2**).

### Extranodal extension

Mean NDSI was significantly higher in ENE-positive disease than ENE-negative disease (86.9% *vs* 43.8%; p<0.001) (**Table 3**). Among ENE-positive patients, ENE >2 mm demonstrated numerically higher NDSI (88.0% *vs* 75.0%); however, this difference was not statistically significant (p=0.429) (**Table 3**).

**Table 3:** NDSI *vs* ENE, and survival analysis.

Variable	Category	N	NDSI (Mean ± SD)	NDSI median (Q1–Q3)	Statistical test	p-value
ENE Status	ENE Negative	55	43.8 ± 30.7	36.7 (17.9–62.0)	Mann–Whitney U test	<0.001
	ENE Positive	49	86.9 ± 20.7	100.0 (80.0–100.0)		
ENE Size*	<2 mm	4	75.0 ± 30.0	80.0 (55.0–100.0)	Mann–Whitney U test	0.429
	≥2 mm	45	88.0 ± 19.8	100.0 (80.0–100.0)		
Survival Status	Alive/Loco-regionally controlled	70	57.2 ± 35.2	55.8 (23.3–100.0)	Mann–Whitney U test	0.004
	Dead	34	78.2 ± 27.1	95.5 (60.0–100.0)		

*ENE size analysis was restricted to the 49 ENE-positive patients.

### Correlation and ROC analysis

NDSI demonstrated a moderate positive correlation with lymph node density (Spearman ρ=0.43, p<0.001) (**Supplementary Table 2**).

**Figure 3:**
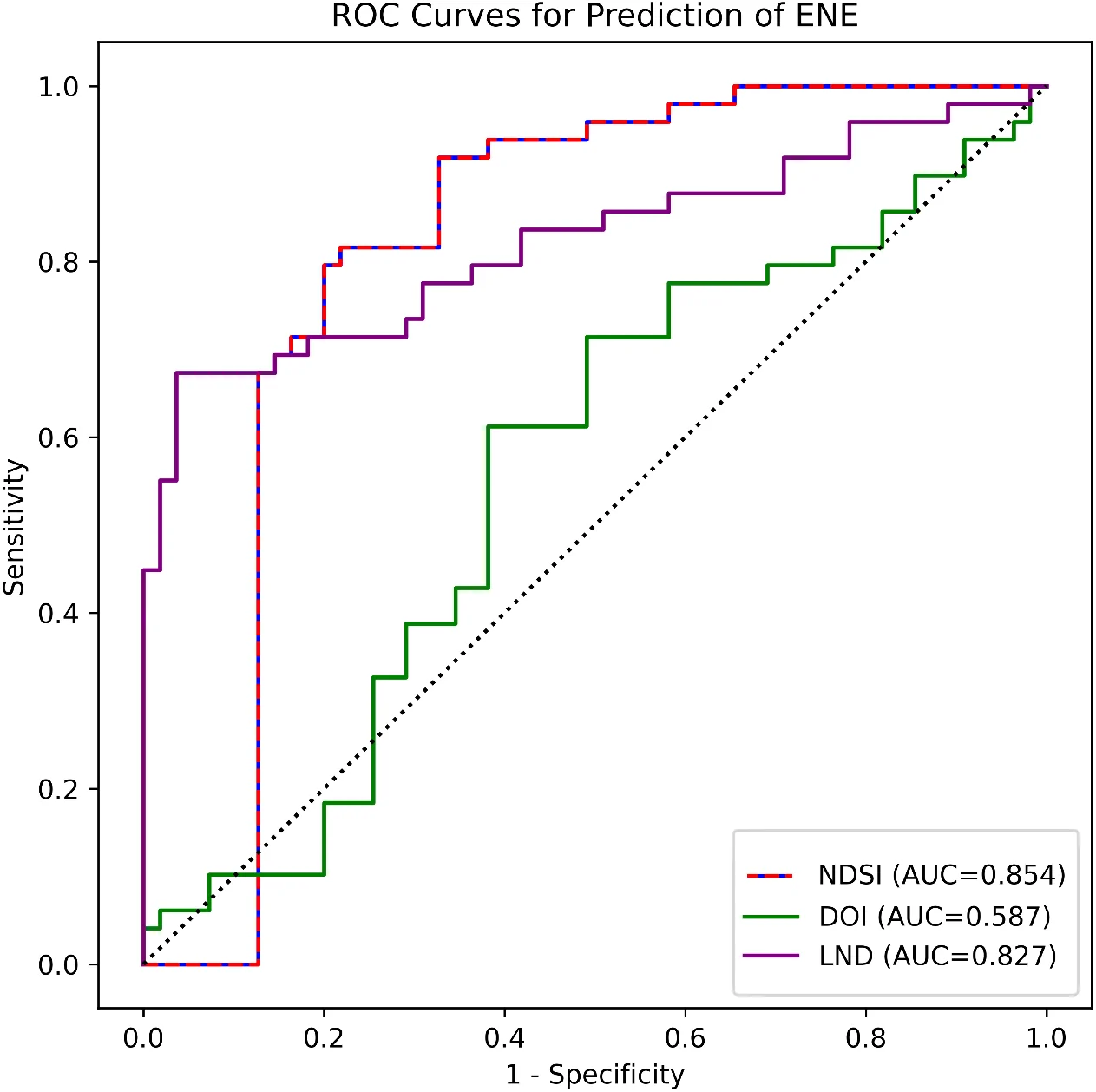
ROC Curves for predicting ENE.

ROC analysis showed an AUC of 0.854 for NDSI in predicting ENE positivity, with an optimal cutoff of 73.3%. LND showed comparable discrimination (AUC = 0.827; cutoff 0.086), whereas DOI showed limited discrimination (AUC = 0.587; cutoff 12 mm). NDSI showed weaker discrimination for ENE ≥2 mm specifically (AUC = 0.667; cutoff 90%) (**Supplementary Table 2**; **Figure 3**).

### Survival analysis

Short term Overall survival was assessed using Kaplan–Meier analysis according to the NDSI cut-off of 73.3%. Of the 104 patients included, 53 had an NDSI <73.3% and 51 had an NDSI >73.3%. During our short term follow-up, 13 deaths (24.5%) occurred in the <73.3% group and 21 deaths (41.2%) in the >73.3% group, while 40 (75.5%) and 30 (58.8%) patients, respectively, were censored.

**Figure 4:**
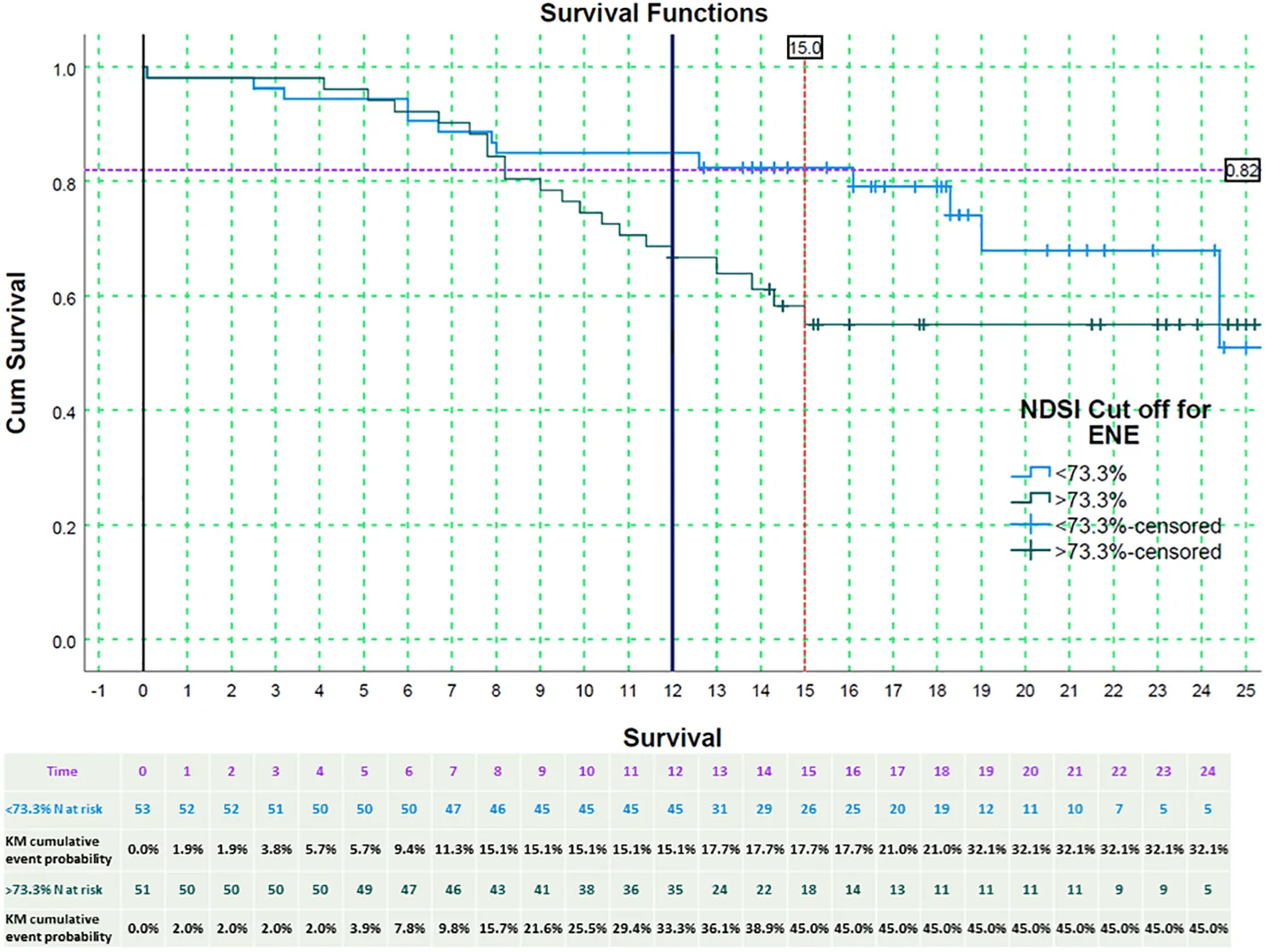
Kaplan-Meier survival curve by NDSI group.

Patients with NDSI >73.3% demonstrated a numerically shorter overall survival compared with those with NDSI <73.3%. The estimated mean survival was 18.13 months (95% CI, 15.78–20.47) in the >73.3% group versus 20.88 months (95% CI, 18.66–23.10) in the <73.3% group. The 75th percentile of survival was 9.9 months in the >73.3% group compared with 18.3 months in the <73.3% group. However, the difference in overall survival between the two groups was not statistically significant on log-rank analysis (χ²=3.049, p=0.081). (**Figure 4**). A separate cutoff of 60% was independently derived via ROC analysis for predicting 1-year mortality (see below); the two cutoffs serve different analytic purposes and should not be conflated as a single recommended threshold. (**Figure 4**).

Patients who died during follow-up demonstrated significantly higher nodal tumour burden than survivors (mean NDSI 78.2% *vs* 57.2%; p=0.004) (**Table 3**).

Higher NDSI correlated with shorter short-term overall survival (Spearman ρ=−0.38, p=0.002) (**Supplementary Table 1**).

ROC analysis demonstrated moderate discrimination of NDSI for predicting death within one year (AUC = 0.708), with an optimal cutoff of 60%, sensitivity of 84.0%, and specificity of 50.6% (**Supplementary Figure 1**).

### Multivariable analysis

The multivariable model was significant (R² = 0.524; adjusted R² = 0.403; F = 4.31; P <0.001). LVI (β = 21.26, 95% CI 2.30–40.22; P = 0.028) and pathological nodal stage were independent predictors of higher NDSI. Compared with pN1 disease, pN2a (β = 33.21, P = 0.001), pN2b (β = 30.44, P = 0.001), and pN3b (β = 47.19, P <0.001) were significantly associated with increased NDSI. WPOI-4 demonstrated a significant inverse association with NDSI (β = −49.52, P = 0.046), whereas WPOI-5 showed a non-significant trend (P = 0.096). Histological differentiation, PPOI, PNI, TILs, NACT, and pathological T category were not independently associated with NDSI (**Table 4**).

**Table 4:** Multivariable analysis with NDSI.

Variable	β coefficient	95% CI	P value
Poorly differentiated	-1.70	-20.54 to 17.13	0.858
Spindle cell SCC	-5.28	-45.62 to 35.06	0.795
Well differentiated	-16.88	-58.74 to 24.99	0.425
WPOI-4	-49.52	-98.17 to -0.87	0.046
WPOI-5	-37.88	-82.59 to 6.83	0.096
PPOI Pattern 4	19.68	-24.77 to 64.13	0.381
PPOI Pattern 5	3.09	-36.24 to 42.42	0.876
PNI (Yes)	-2.99	-20.87 to 14.89	0.740
LVI (Yes)	21.26	2.30 to 40.22	0.028
NACT (Yes)	2.46	-14.39 to 19.31	0.772
T1	-24.98	-97.08 to 47.13	0.493
T2	-27.70	-102.92 to 47.52	0.466
T3	-28.31	-100.85 to 44.23	0.440
T4a	-18.91	-92.84 to 55.03	0.612
T4b	-5.78	-87.98 to 76.42	0.889
pN2a	33.21	13.17 to 53.26	0.001
pN2b	30.44	13.14 to 47.73	0.001
pN2c	-26.40	-84.42 to 31.62	0.368
pN3a	62.73	-6.26 to 131.73	0.074
pN3b	47.19	31.76 to 62.61	<0.001
TILs (%)	-0.22	-0.58 to 0.14	0.220

## Discussion

Current pathological nodal staging systems rely predominantly on nodal number, laterality, size, and extranodal extension. Although these variables provide prognostic information, they do not directly quantify the degree of metastatic replacement within the lymph node (8). Two lymph nodes with similar maximum diameters may contain different proportions of tumour and preserved nodal architecture. NDSI was developed to quantify this relationship using routine histopathological measurements. Metastatic deposits within lymph nodes have been evaluated in colorectal carcinoma (9), malignant melanoma (10) and breast cancer (11).

The concept of NDSI evolved from the staging relevance of micrometastases and isolated tumour cells, where the extent of metastatic deposit within lymph nodes influences prognosis. NDSI was developed to quantify metastatic replacement of nodal architecture and the residual uninvolved nodal component. This extends the concept of micrometastatic disease from categorical detection to quantitative assessment of intranodal tumour burden in OSCC (6).

Increasing metastatic occupancy may reflect tumour expansion within the nodal microenvironment and progressive disruption of residual lymphoid architecture; this remains a biological hypothesis rather than a finding directly tested in the present study, which evaluated only linear morphometric measurements. Because ENE positivity directly determines pN3b classification under AJCC 8th edition criteria in several nodal categories, the association between NDSI and pathological N-stage is not fully independent of the NDSI-ENE association, and the two findings should not be interpreted as separate lines of evidence. The principal contribution of NDSI independent of composite staging therefore lies in its association with ENE and survival at the level of the individual node, rather than at the level of stage, which by definition already incorporates ENE status.

Prior studies have evaluated nodal burden using nodal yield, LNR, and LND, both of which have shown prognostic relevance in oral cavity and laryngeal cancers (12–14). However, both metrics are influenced by extent of neck dissection, pathological nodal yield, and specimen handling (15). In contrast, NDSI evaluates the metastatic lymph node directly and remains independent of total nodal harvest.

The stepwise increase in NDSI across both pathological N category and overall stage is consistent with progressive replacement of nodal architecture by metastatic tumour as disease advances, though as noted above this parallels rather than independently confirms the established prognostic weight of stage and ENE.

Lymphovascular invasion and perineural invasion were significantly associated with increased NDSI, consistent with prior reports linking these features to more extensive nodal involvement (16).

An inverse relationship was observed between TIL density and NDSI, with low-TIL tumours showing higher nodal tumour occupancy and lower residual nodal reserve (17, 18). Although detailed immune profiling was not performed, this single non-significant trend should be interpreted with caution and is presented as a hypothesis for future investigation rather than as evidence of an immune-metastatic interaction.

NDSI demonstrated a moderate correlation with LND (ρ=0.43), indicating partial but incomplete overlap between the two parameters. LND reflects the proportion of metastatic lymph nodes among all excised nodes (19) and therefore represents the extent of regional nodal dissemination, whereas NDSI quantifies the proportion of an individual metastatic lymph node occupied by tumour, reflecting intranodal tumour burden.

WPOI and PPOI demonstrated numerical trends toward increasing NDSI with more aggressive invasive patterns but did not achieve statistical significance. This may relate to subgroup sample size limitations and missingness within selected pathological categories. WPOI has been reported as a predictor of nodal metastasis in prior studies (20).

DOI similarly demonstrated numerically increasing NDSI with increasing DOI groups, although statistical significance was not reached. DOI has been reported as a predictor of nodal metastasis in OSCC (21). The absence of statistical significance in the present analysis may reflect cohort size and subgroup variability.

We also observed a significant association between the highest involved neck level and NDSI. However, unlike pathological N stage, no consistent stepwise anatomical trend was evident across neck levels, and several subgroups contained relatively few patients. Consequently, this finding should be interpreted cautiously and considered hypothesis-generating until validated in larger cohorts.

Although WPOI, PPOI and DOI are established adverse pathological markers in OSCC and have been associated with nodal metastasis and poorer oncological outcomes, none demonstrated an independent association with NDSI in the present study. This likely reflects the relatively small cohort, imbalance across pathological subgroups, and limited statistical power rather than absence of a biological relationship. Furthermore, these parameters primarily characterize the invasive behaviour of the primary tumour, whereas NDSI quantifies metastatic tumour occupancy within involved lymph nodes. These findings suggest that NDSI may represent a distinct aspect of tumour biology rather than simply mirroring established markers of primary tumour aggressiveness.

Inferior short-term survival among ENE-positive patients in this cohort is consistent with prior reports (22–24). Patients with NDSI >73.3% showed a trend toward inferior overall survival during short-term follow-up, although statistical significance was not achieved on Kaplan–Meier analysis. Limited follow-up duration, cohort size, and heterogeneity of primary subsites may have influenced this finding.

The ROC-derived AUC for 1-year mortality (0.708) should not be interpreted as evidence of prognostic significance independent of the non-significant Kaplan–Meier comparison reported above; ROC and log-rank analyses test related but distinct hypotheses, and the discordance between a moderate AUC and a non-significant survival difference likely reflects the limited number of events (34 deaths) rather than a true predictive signal. As this cutoff was derived and evaluated within the same cohort without internal validation (e.g., bootstrapping or split-sample testing), it is also subject to optimism bias, and the reported AUC may not be reproducible in an independent cohort.

Multivariable analysis demonstrated that LVI and pathological nodal stage were independent determinants of higher NDSI. Patients with pN2a, pN2b, and pN3b disease had significantly greater NDSI than those with pN1 disease, indicating that increasing nodal stage is associated with a higher proportion of nodal replacement by metastatic tumour. Other clinicopathological variables, including differentiation, PPOI, PNI, TILs, NACT, and pathological T category, were not independently associated with NDSI after adjustment for confounding variables. WPOI demonstrated limited independent significance, likely reflecting overlap with other adverse pathological features. Although PNI demonstrated a significant association with NDSI on univariable analysis, this association did not persist after multivariable adjustment, suggesting that its effect may be mediated through coexisting adverse pathological features, particularly nodal stage and LVI.

Since NDSI incorporates metastatic deposit size while accounting for the overall LyND, it differs conceptually from metastatic deposit size alone. The present study was not designed to directly compare the prognostic performance of NDSI with metastatic deposit diameter alone.

The study has several limitations. It was a single-centre study with a relatively small cohort of 104 node-positive patients and no external validation, limiting statistical power, particularly for subgroup and survival analyses. Consequently, some analyses may have been underpowered, and non-significant findings should not be interpreted as absence of association. Selected pathological variables (WPOI, PPOI, and DOI) had missing data, and several subgroups (e.g., pT4b, pN2c, well-differentiated and spindle cell SCC) contained fewer than five patients. These subgroups remain included in the omnibus Kruskal–Wallis tests reported in **Tables 1**, **2** for completeness, but their contribution to the test statistic is limited and findings involving them should be interpreted descriptively rather than inferentially. Potential confounders including smoking and alcohol exposure, margin status, and receipt of adjuvant therapy were not incorporated into this analysis and may independently influence both nodal tumour burden and survival. Multiple exploratory subgroup analyses were performed without adjustment for multiplicity; therefore, statistically significant findings should be considered hypothesis-generating. The ambispective design also introduces the potential for selection and information bias. This study is reported in accordance with the STrengthening the Reporting of OBservational studies in Epidemiology (STROBE) statement for cohort studies.

Survival analyses should be interpreted cautiously because follow-up was relatively short, precluding assessment of long-term oncological outcomes. Morphometric assessment was based on two-dimensional linear measurements rather than volumetric analysis. NDSI should not be interpreted as a direct measure of tumour area or volume within a lymph node. Rather, it represents a pragmatic linear morphometric surrogate derived from routine histopathological measurements that was intentionally designed for simplicity and reproducibility in routine pathology practice. Furthermore, interobserver reproducibility was not evaluated and requires formal validation before routine clinical implementation.

The limited number of ENE-positive cases and survival events precluded adequately powered multivariable logistic regression for ENE and Cox proportional hazards modelling for overall survival. These analyses should be evaluated in larger prospective cohorts.

Importantly, NDSI is not intended to replace AJCC nodal staging or ENE. Rather, they quantify a complementary aspect of nodal disease by estimating the extent of metastatic replacement within an involved lymph node. Future multicentre studies with larger cohorts, longer follow-up, external validation, reproducibility assessment, and comparison with volumetric and molecular biomarkers are required before clinical adoption.

## Conclusion

NDSI is a histomorphometric measures derived from routine pathological assessment that correlate with adverse pathological characteristics and early survival outcomes in node-positive oral squamous cell carcinoma. These findings are hypothesis-generating. Validation in larger multicentre cohorts with longer follow-up, assessment of interobserver reproducibility, internal and external validation of the proposed cutoffs, and clarification of incremental prognostic value independent of AJCC nodal staging and ENE are required before any consideration of clinical application.

## Data Availability

The raw data supporting the conclusions of this article will be made available by the authors, without undue reservation.

## Supplementary material

Supplementary Figure 1 ROC Curve for death in 1 year – NDSI.

Supplementary Table 1 NDSI survival correlation.

Supplementary Table 2 Correlation and ROC analysis for prediction of ENE positivity.
