# Supplementary material for: Node deposit size index as morphometric marker of intranodal tumour burden in node-positive oral cancer: a hypothesis-generating cohort study
Source: Front Oncol. 2026 Sep 7;16:1923931. doi: 10.3389/fonc.2026.1923931 (PMC13590355; doi:10.3389/fonc.2026.1923931)
Supplement: Supplementary file 1 [file Supplementaryfile1.docx]

*Supplement figure 1: ROC Curve for death in 1 year - NDSI*

### *Supplement Table 1: NDSI Survival Correlation*

| Parameter | Analysis Type | Result | p-value |
| --- | --- | --- | --- |
| NDSI vs survival duration | Spearman correlation | rho = -0.382 | 0.002 |

*Supplement Table 2: Correlation and ROC Analysis for Prediction of ENE Positivity*

| Parameter | Analysis Type | Result | p-value | Optimal Cut-off | AUC |
| --- | --- | --- | --- | --- | --- |
| NDSI vs LND | Spearman correlation | ρ = 0.43 | <0.001 | — | — |
| NDSI predicting ENE positivity | ROC analysis | — | — | 73.3% | 0.854 |
| DOI predicting ENE positivity | ROC analysis | — | — | 12 mm | 0.587 |
| LND predicting ENE positivity | ROC analysis | — | — | 0.086 | 0.827 |
| NDSI predicting ENE ≥2 mm | ROC analysis | — | — | 90.0% | 0.667 |
